# Expression of fibroblast growth factor receptor 2 (FGFR2) in combined hepatocellular-cholangiocarcinoma and intrahepatic cholangiocarcinoma: clinicopathological study

**DOI:** 10.1007/s00428-024-03792-x

**Published:** 2024-03-27

**Authors:** Motoko Sasaki, Yasunori Sato, Yasuni Nakanuma

**Affiliations:** 1 Department of Human Pathology, Kanazawa University Graduate School of Medical Sciences, Kanazawa, 920-8640 Japan; 2 Division of Pathology, Fukui Saiseikai Hospital, Fukui, Japan

**Keywords:** FGFR2, Genetic alterations, Combined hepatocellular and cholangiocarcinoma, Intrahepatic cholangiocarcinoma-small duct type, BAP1, Nestin

## Abstract

Genetic alterations including fusions in fibroblast growth factor receptor 2 (FGFR2) are detected in 10–20% of intrahepatic cholangiocarcinoma (iCCA), and FGFR2 inhibitors are effective for the treatment of iCCA. We examined a prevalence of FGFR2 genetic alterations and their clinicopathological significance in combined hepatocellular–cholangiocarcinoma (cHCC-CCA). FGFR2 expression, which is a surrogate marker for FGFR2 genetic alterations, was immunohistochemically assessed in the liver sections from 75 patients with cHCC-CCA, 35 with small duct-type iCCA, 30 with large duct-type iCCA, and 35 with hepatocellular carcinoma (HCC). FGFR2 genetic alterations were detected by reverse transcription-PCR and direct sequence. An association of FGFR2 expression with clinicopathological features was investigated in cHCC-CCAs. FGFR2 expression was detected in significantly more patients with cHCC-CCA (21.3%) and small duct-type iCCA (25.7%), compared to those with large duct-type iCCA (3.3%) and HCC (0%) (*p* < 0.05). FGFR2-positive cHCC-CCAs were significantly smaller size (*p* < 0.05), with more predominant cholangiolocarcinoma component (*p* < 0.01) and less nestin expression (*p* < 0.05). Genetic alterations of ARID1A and BAP1 and multiple genes were significantly more frequent in FGFR2-positive cHCC-CCAs (*p* < 0.05). 5′/3′ imbalance in FGFR2 genes indicating exon18-truncated FGFR2 was significantly more frequently detected in FGFR2-positive cHCC-CCAs and small duct iCCAs, compared to FGFR2-negative ones (*p* < 0.05). *FGFR2::BICC* fusion was detected in a case of cHCC-CCAs. FGFR2 genetic alterations may be prevalent in cHCC-CCAs as well as small duct-type iCCAs, which suggest cHCC-CCAs may also be a possible therapeutic target of FGFR2 inhibitors.

**Graphical Abstract:**

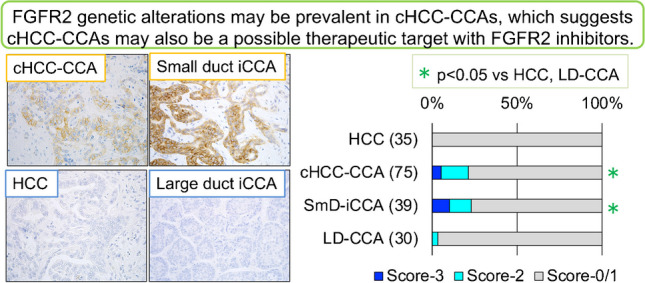

**Supplementary Information:**

The online version contains supplementary material available at 10.1007/s00428-024-03792-x.

## Introduction

Combined hepatocellular-cholangiocarcinoma (cHCC-CCA), which generally has a poor prognosis, comprises of hepatocellular carcinoma (HCC), cholangiocarcinoma (CCA), and diverse components with intermediate features between HCC and CCA [1–3]. The histological diagnosis of cHCC-CCA is sometimes difficult and controversial because of intratumoral heterogeneity with diverse intermediate components [1–3]. A consensus paper has provided simplified terminology and refined the diagnostic criteria for cHCC-CCA [3], and the current WHO classification 2019 adopted this consensus [1]. The histopathological diagnosis of cHCC-CCA needs to be standardized for the appropriate clinical treatment of patients [2]. Previous studies disclosed that some cHCC-CCAs had similar genetic alterations to HCCs, whereas other cHCC-CCAs had similar genetic alterations to CCAs [4–7]. The genetic alterations and other molecular features in cHCC-CCAs may be therapeutic targets, as well as HCCs and CCAs [2, 4–7].

Accumulating data suggest that about a half of iCCAs have targetable genetic alterations [8, 9]. The fibroblast growth factor receptor 2 (FGFR2), which is one of four FGFR family members that encode transmembrane receptor tyrosine kinases, has attracted much attention [10–14]. The FGFR2 fusions or rearrangements are found as genetic abnormalities in 10–20% of iCCA, especially in small duct-type iCCAs [10–12, 15]. Over 150 fusion partners are detected in FGFR2 fusion genes [12, 16], and a recent study revealed the truncation of exon 18 (E18) of FGFR2 is a potent driver mutation and could be a therapeutic target [17]. Immunohistochemical FGFR2 expression may be a candidate surrogate marker for detecting FGFR2 genetic alterations with high specificity and a prognostic marker in iCCA [18, 19]. FGFR2 inhibitors, such as pemigatinib and futibatinib, inhibit tumor cell growth in FGFR‐driven cancers by receptor autophosphorylation and subsequent activation of FGF/ FGFR signaling [20]. Favorable therapeutic effects of these FGFR inhibitors are observed in several clinical trials in iCCAs [16, 21].

cHCC-CCAs shares various features such as histological findings of iCCA components, etiologies, and possible cell origin with small duct-type iCCA [1, 2, 22]; however, there were only a few studies on FGFR2 genetic alterations in cHCC-CCA, so far [4–7]. In previous studies, FGFR2-fusions were detected in 0–6.5% of cHCC-CCAs, and the prevalence was higher in CCA-like cHCC-CCAs compared to HCC-like ones [4]. We examined a prevalence of FGFR2 genetic alterations and its clinicopathological significance in cHCC-CCA in this study. We took advantage of an immunostaining for FGFR2 as a surrogate marker and then performed the fusion-specific PCR with following direct sequencing and 5′/3′ imbalance PCR [23] for the detection of exon 18 (E18)-truncated FGFR2 including FGFR2 fusions [17]. There has been no study on the immunohistochemical expression of FGFR2 in cHCC-CCAs, to our knowledge.

## Materials and methods

### Patients and preparation of tissue specimens

One hundred and seventy-nine patients with primary liver carcinoma were retrieved from our pathological files (1996–2022). The Ethics Committee of Kanazawa University approved the present study (The approval number: 2012–021 [160]; the date, June 11, 2013). Primary liver carcinomas were re-evaluated according to the WHO classification of digestive system tumors 2019 and classified into 75 with cHCC-CCA, 35 with small duct-type iCCA, 30 with large duct-type iCCA, and 35 with hepatocellular carcinoma (HCC). A diagnosis of cHCC-CCA was made regardless of the percentage of each component in the present study [1]. Cholangiolocarcinoma/cholangiolocellular carcinoma (CLC) was classified into small duct-type iCCA as a subtype in the present study, according to the WHO classification of digestive system tumors 2019 [22]. Clinical and pathological features in each group of primary liver carcinomas are summarized in Table 1.

**Table 1:** Clinicopathological features of primary liver carcinoma examined

	HCC	Combined HCC-iCCAs	iCCA, Small duct type	iCCA, Large duct type	*p*
Number of patients	35	75	35	30	
*Age* mean (y; range)	65.4 (39–86)	65.0 (36–83)	69.7 (52–83)	63.4 (39–84)	ns
*Gender* (male, %)	32 (91.4%)	51 (68%)*	26 (74.3%)^#^	16 (53.3%)*	*, *p* < 0.01, #, *p* < 0.05 vs HCC
*Etiology*					
B/C/alcohol/NAFLD/?	10/14/6*^a^/3/2	23/26/5*^a^ /6/16	1/7/6*^a^/2/20	2/2/0/1/25	
* Virus (B or C)*	24 (68.6%) *	49 (65.3%) *	8 (22.9%)	4 (13.3%)	*, *p* < 0.01, vs iCCA, large duct and iCCA, small duct
Positive (%)					
* Alcohol and/or NAFLD*	9 (25.7%) *	12 (13.3%)	8 (22.9%)	2 (6.7%)	*, *p* < 0.05, vs iCCA, large duct
Positive (%)					
*Fibrosis* F3,4/F1,2/0	28/6/1	55/14/6	12/8/15	1/2/27	
* F3,4 *(%)	28 (80%) *	55 (73.3%) *	12 (34.3%) *	1 (3.3%)	*, *p* < 0.01, vs iCCA, large duct
*Tumor size* mean (mm, range)	31.4 (12–130)	40.4 (5–130)	34.1 (10–130)	51.1* (18–100)	*, *p* < 0.01: vs HCC
*Previous therapy* positive (%)	3 (8.6%)	21 (28.0%) *	0	0	*, *p* < 0.01, vs iCCA, large duct and iCCA, small duct
*Multiple tumors* positive (%)	9 (25.7%) *	23 (30.7%) *	6 (17.1%)	0	*, *p* < 0.05 vs iCCA, large duct

*HCC*, hepatocellular carcinoma; *iCCA*, intrahepatic cholangiocarcinoma; *B*, hepatitis B; *C*, hepatitis C; *NAFLD*, nonalcoholic fatty liver disease; ?, others/unknown; *F0-4*, degree of fibrosis according to Shin-Inuyama classification. *a, a patient with hepatitis B and alcohol

All specimens were surgically resected and fixed in 10% buffered formalin and embedded in paraffin. Three-micrometer-thick sections were cut from each paraffin block. Several sections were routinely processed for histological studies, including hematoxylin and eosin stain, reticulum stain, AZAN, and mucin staining, and the remainder were processed for the following immunohistochemistry.

### Histological grading and the ductal plate malformation (DPM) pattern

The histological grading of cHCC-CCA was classified into low and high grades being based on tumor differentiation [24]. The DPM-pattern was evaluated as previously described [25]. The DPM-pattern was characterized by neoplastic glands of carcinoma showing an irregularly shaped and dilated lumen, and some of these glands showed microcystic dilatation, resembling DPM. The degree of the DPM-pattern was divided into absent (< 5% of the tumor), focal (5–50%), and extensive (> 50%). Among the 75 cHCC-CCA, 47, 21, and 7 showed the absent, focal, and extensive patterns, respectively.

### Immunohistochemistry

The expression of FGFR2, ARID1A, p53, PBRM1, BAP1, MTAP, and nestin was examined by immunostaining, as previously described [26, 27]. The primary antibodies used are shown in Supplementary Table 1. Positive and negative controls were routinely included.

#### Evaluation of immunostaining for FGFR2

The expression of FGFR2 in cell membrane was evaluated as described previously: score 3, strong, complete membrane staining in more than 10% of the malignant cells; score 2, weak to moderate, complete membrane staining in more than 10% of the malignant cells; and score 0/1, less intense staining or less than 10% of cells, according to previous study [19]. A score of 2 or 3 was considered positive and scores of 0 or 1 were considered negative (two‐grade system).

#### Evaluation of immunostaining for p53

Strong and diffuse nuclear expression was regarded as a mutation of p53, as previously described [26]. Three patterns of aberrant or mutation-type p53 staining that are indicative of an underlying *p53* mutation, including overexpression (strong nuclear staining in at least 75% of tumor cells), the null pattern (loss of staining in 100% of tumor cells), and the cytoplasmic pattern, were demonstrated in previous studies [28, 29]. Accordingly, the null and cytoplasmic patterns were also examined, but not observed in any specimens in the present study.

#### Evaluation of immunostaining for ARID1A, PBRM1, and BAP1

The total or focal loss of nuclear expression was regarded as a genetic alteration. Total and focal loss of expression was observed. When the expression was totally lost in the tumor, the specimen was regarded as “total loss,” whereas the expression was lost in a part of the tumor, the specimen was regarded as “focal clonal loss.” It has been reported as a reliable marker for inactivating genetic alterations in ARID1A, PBRM1, and BAP1; however, the immunostaining was not affected by some missense mutations [30–32].

#### Evaluation of immunostaining for MTAP

MTAP loss in immunohistochemistry is reportedly a reliable surrogate for *CDKN2A* homozygous deletion [33]. Loss of cytoplasmic expression of MTAP was regarded as *CDKN2A* homozygous deletion. Total and focal loss of expression was observed [33].

#### Evaluation of immunostaining for nestin

The expression of nestin (diffuse cytoplasmic) was evaluated according to the percentage of positive cells in each lesion: score 0, less than 5%; score 1, 5–10%; score 2, 10–80%; score 3, more than 80%. Scores 1–3 were regarded as positive. Inter-observer agreement was almost perfect in the present study.

### Extraction of RNA samples and assessment of FGFR2 genetic alterations

Twenty-four cHCC-CCAs and 9 small duct-type iCCAs were examined for FGFR2 genetic alterations using PCR and direct sequence. Representative whole sections which include both HCC and iCCA components to various degree were used for the extraction of RNA in each case. RNA samples were extracted from FFPE sections using RNeasy-FFPE kit (QIAGEN, Hilden, Germany), and then cDNA samples are made using Quant Accuracy RT-RamDA cDNA Synthesis Kit (TOYOBO, Osaka, Japan) according to manufactures’ protocols.

#### Detection of FGFR2 fusions

PCR was performed using FGFR2-fusion-specific primers (Supplementary Table 2). Direct sequences of PCR products were performed as described previously [27].

#### 5′/3′ imbalance strategy for the detection of exon 18 (E18)-truncated FGFR2

E18-truncated FGFR2 including the FGFR2 fusion genes [17] were detected by measuring the ratio of the expression levels of 5′ portion (exon 5, E5) versus the 3′ portion (E18) of the FGFR2 expression using the Thunderbird qPCR Master Mix (Toyobo, Tokyo, Japan) and the QuantStudio 6 Pro real-time PCR system (Thermo-Fisher, Waltham, USA) according to manufactures’ protocol. The PCR primers used were shown in Table 3. This 5′/3′ imbalance strategy was developed, with high specificity and sensitivity, for detection of the ALK fusion gene [23]. In the presence of the E18-truncation in FGFR2 gene including FGFR2 fusion gene, the 3′ portion of the FGFR2 gene (E18) is lost, but the 5′ portion (E5) remains. This strategy could effectively detect the E18-truncated FGFR2 gene no matter which partner genes were at the 3′ portion of the fusion genes.

### Extraction of DNA samples and mutation analysis of KRAS, IDH1, IDH2, and the TERT promoter

The extraction of DNA samples, PCR and sequencing were performed as previously described [26]. The primer sets for PCR are shown in Table 3.

### Statistical analysis

The Kruskal–Wallis test was used for continuous variables without a normal distribution. If a significant difference was observed in an analysis of variance, pairwise comparisons were performed using Dunn’s post hoc test with corrections for multiple comparisons. When the *p* value was less than 0.05, the difference was considered as significant. All analyses were performed using the GraphPad Prism software (GraphPad Software, San Diego, CA, USA).

## Results

### FGFR2 expression in primary liver carcinoma and the background liver

**Fig. 1:**
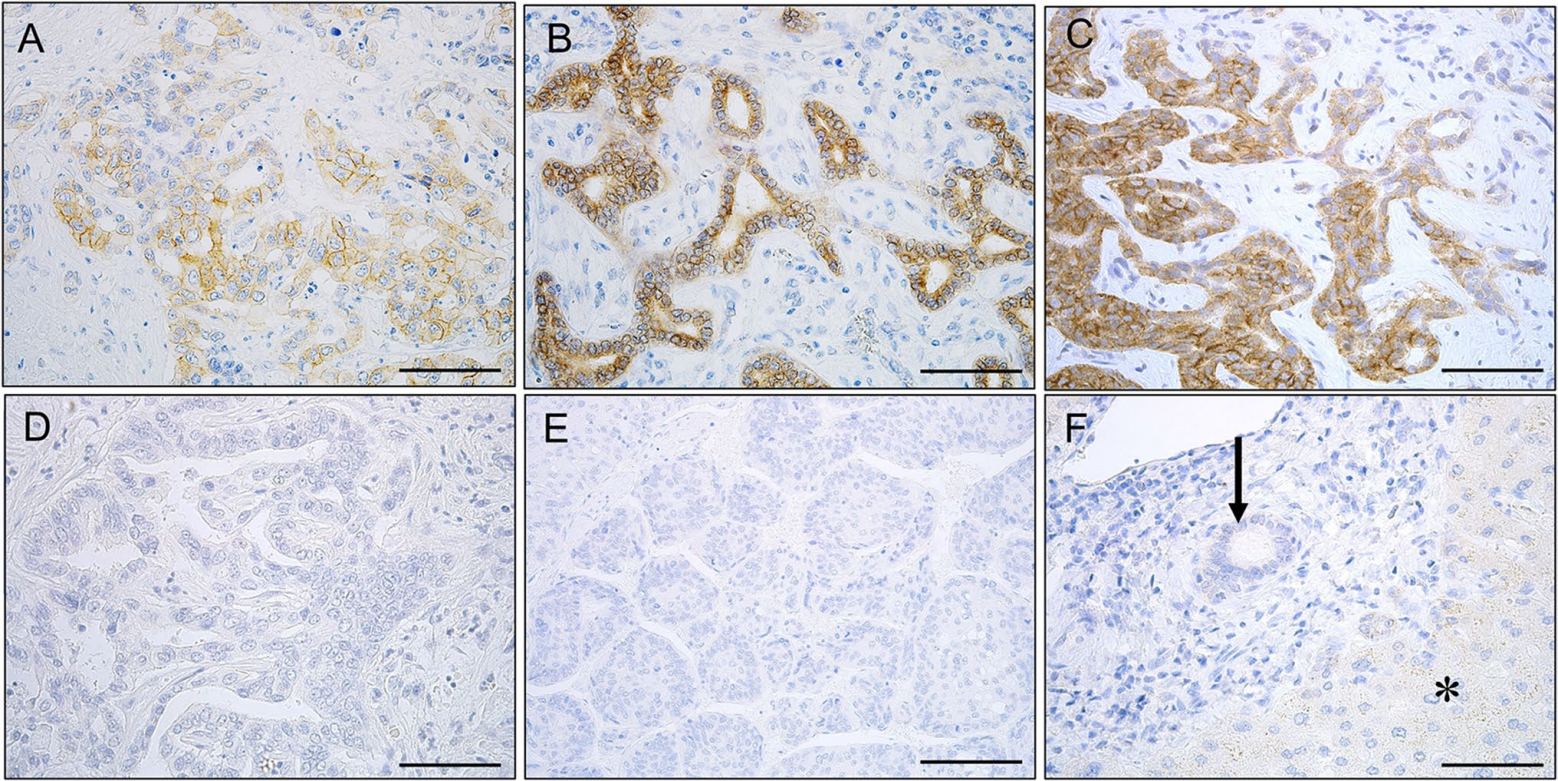
Expression of FGFR2 in primary liver carcinoma. **A** An example of combined hepatocellular-cholangiocarcinoma (cHCC-CCA) showing FGFR2 expression. FGFR2 is expressed in the cell membrane of carcinoma cells in cHCC-CCA. FGFR2-score 3. A 44-year-old female, glycogen storage disease type I, a tumor size of 7 cm, F4. **B** An example of cHCC-CCA showing FGFR2 expression. FGFR2 is expressed in the cell membrane of carcinoma cells in cHCC-CCA. FGFR2-score 3. A 63-year-old male, cHCC-CCA with the cholangiolocarcinoma (CLC) component, hepatitis B and alcohol, a tumor size of 1.9 cm, F3. **C** An example of small duct-type intrahepatic cholangiocarcinoma (iCCA) showing FGFR2 expression. FGFR2 is expressed in the cell membrane of carcinoma cells. FGFR2-score 3. A 78-year-old female, negative for hepatitis B and C, a tumor size of 1.5 cm, F1. **D** An example of large duct-type iCCA without FGFR2 expression. FGFR2 is not expressed in carcinoma cells in large duct-type iCCA. A 73-year-old female, hepatitis B, a tumor size of 2.6 cm, F1. **E** FGFR2 is not expressed in carcinoma cells in hepatocellular carcinoma. A 62-year-old male, hepatitis B, a tumor size of 5.5 cm, F3. **F** FGFR2 is not expressed in the non-neoplastic bile duct (arrow) or hepatocytes (asterisk) in the background liver. Immunostaining for FGFR2, counterstained by hematoxylin. Scales are 50 μm

Figure 1 shows examples of FGFR2 expression in cHCC-CCAs and other types of primary liver carcinomas and the background livers. Supplementary Fig. 1 shows examples of histology in cHCC-CCAs and small duct iCCA showing FGFR2 expression. FGFR2 was expressed in the cell membrane of carcinoma cells if present. FGFR2 was not expressed in non-neoplastic bile ducts or hepatocytes (Fig. 1). The expression of FGFR2 was observed in a part of cHCC-CCAs and small duct-type iCCAs (Fig. 1). The expression of FGFR2 was detected in one large duct-type iCCAs and none of HCCs. The expression of FGFR2 was detected in significantly more patients with cHCC-CCAs (21.3%) and small duct-type iCCAs (25.7%), compared to those with large duct-type iCCAs (3.3%) and HCCs (0%) (*p* < 0.05) (Table 2).

**Table 2:** Expression of fibroblast growth factor receptor 2 (FGFR2) in primary liver carcinoma

	Number of patients	*FGFR2* positive (%)	FGFR2 score [3, 2, 0/1]	*p*
HCC	35	0 (0%)	0/0/35	
Combined HCC-iCCAs	75	16 (21.3%)*,#	4/12/59	*, *p* < 0.01, vs HCC #, *p* < 0.05, iCCA, large duct type
iCCA, small duct type	35	9 (25.7%)*,#	4/5/26	*, *p* < 0.01, vs HCC #, *p* < 0.05, iCCA, large duct type
iCCA, large duct type	30	1 (3.3%)	0/1/29	

*HCC*, hepatocellular carcinoma; *iCCA*, intrahepatic cholangiocarcinoma; *CLC*, cholangiolocellular carcinoma; *, *p* < 0.01, vs HCC

^#^, *p* < 0.05, iCCA, large duct type

### Relationships between FGFR2 expression and clinicopathological features in cHCC-CCA

Table 3 summarizes the association of FGFR2 expression with clinicopathological features and genetic alterations in 75 patients with cHCC-CCA. FGFR2-positive cHCC-CCAs were significantly smaller size (*p* < 0.05), with more predominant cholangiolocarcinoma component (*p* < 0.05) and less nestin expression (*p* < 0.05), compared to FGFR2-negative cHCC-CCAs. Genetic alterations of ARID1A and BAP1 and multiple genetic alterations were significantly more frequent in FGFR2-positive cHCC-CCAs, compared to FGFR2-negative cHCC-CCAs (*p* < 0.05).

**Table 3:** Relationships between fibroblast growth factor receptor 2 (FGFR2) expression and clinicopathological features and genetic alterations in combined hepatocellular-cholangiocarcinoma

	FGFR2 positive (16)	FGFR2 negative (59)	*p*-value
*Age* mean (y; range)	69.3 (36–82)	66.3 (44–79)	ns
*Gender* (male, %)	9 (56%)	42 (71%)	ns
*Etiology* B/C/alcohol/NAFLD/unknown	8/5/0/0/3	16/19/5/6/13	
	B, C: 81%	B, C: 59%	ns
	Alcohol, NAFLD: 0%	Alcohol, NAFLD: 19%	ns
*Fibrosis*	10/6	46/13	ns
F3,4/F0-2	F3,4: 63%	F3,4: 78%	
*Tumor size* mean (mm, range)	31 (5–87)	43 (20–130)	*p* < 0.05
*Previous therapy* positive (%)	2 (13%)	17 (29%)	ns
*Multiple tumors* positive (%)	5 (31%)	17 (29%)	ns
*Histological grade* low/high	4/12	14/45	ns
*CLC component* positive (%)	12 (75%)	44 (75%)	ns
*CLC component* > *80% (%)*	6 (38%)	7 (12%)	*p* < 0.05
*DPM-like pattern* (2/1/0)	3/6/7	4/15/40	ns
*AFP* positive (%)	7 (44%)	21 (36%)	ns
*Nestin* positive (%)	7 (44%)	43 (73%)	*p* < 0.05
*ARID1A alteration (%)*	5 (31%)	5 (8%)	*p* < 0.05
*p53 alteration (%)*	10 (63%)	29 (49%)	ns
*PBRM1 alteration (%)*	2 (13%)	12 (20%)	ns
*BAP1 alteration (%)*	3 (19%)	1 (2%)	*p* < 0.01
*KRAS alteration (%)*	0	4 (7%)	ns
*IDH1/2 alteration (%)*	0	5 (9%)	ns
*hTERT promoter mutation (%)*	2 (13%)	20 (34%)	ns
*MTAP alteration (%)*	3 (19%)	10 (17%)	ns
*Multiple genetic alterations (%)*	15 (94%)	26 (44%)	*p* < 0.01
*Any alterations (%)*	16 (100%)	48 (81%)	ns

*B*, hepatitis B; *C*, hepatitis C; *NAFLD*, nonalcoholic fatty liver disease; ?, others/unknown; *F0-4*, degree of fibrosis according to Shin-Inuyama classification; *CLC*, cholangiolocarcinoma; *DPM*, ductal plate malformation; *AFP*, α-fetoprotein; *ARID1A*, AT-rich interactive domain-containing protein 1A; *PBRM1*, protein polybromo-1; *BAP1*, breast cancer 1 associated protein 1; *hTERT*, human telomerase reverse transcriptase; *MTAP*, methylthioadenosine phosphorylase; *ns*, not significant

### Detection of FGFR2 fusions

**Fig. 2:**
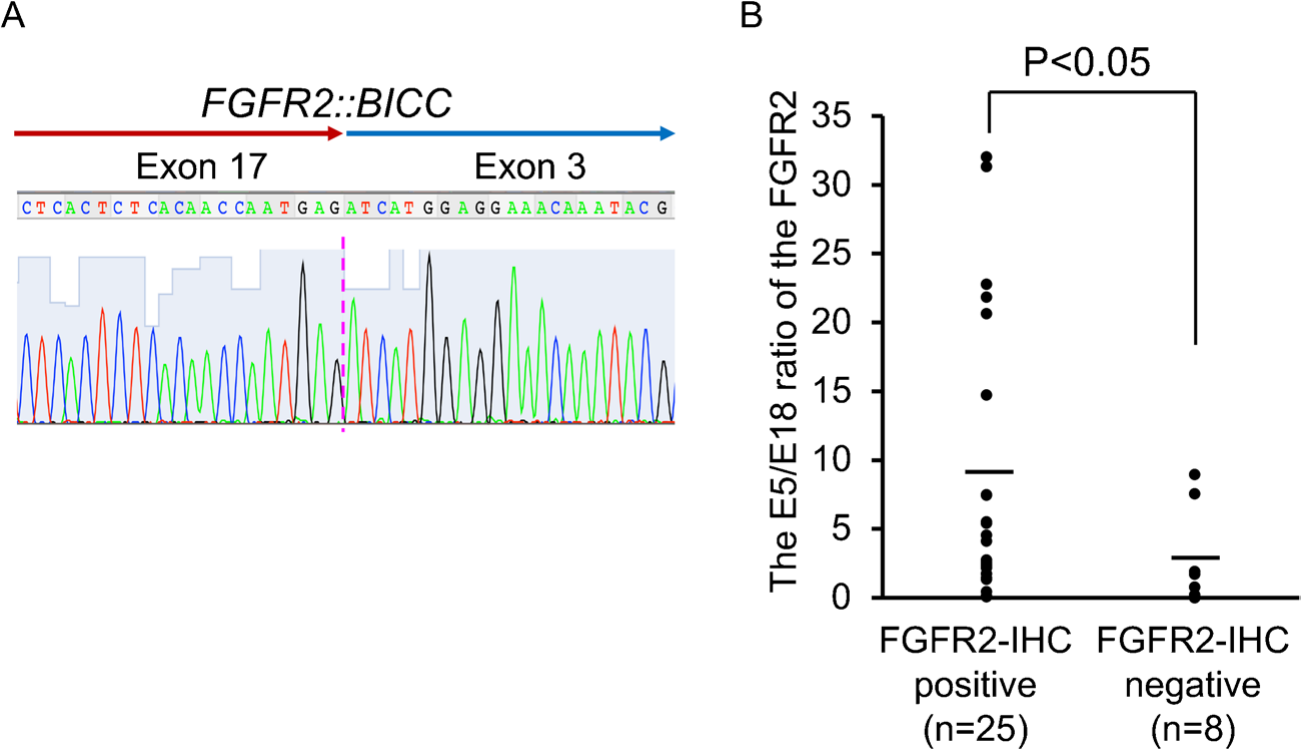
Detection of *FGFR2* fusions and the exon18 (E18)-truncated *FGFR2* in combined hepatocellular-cholangiocarcinoma (cHCC-CCA) and small duct-type intrahepatic cholangiocarcinoma (iCCA). **A** Detection of FGFR2 fusions. Schematic representation of the identified *FGFR2* fusion genes. Sanger sequencing confirming the chimeric junction between *FGFR2* and *BICC1* in a case of cHCC-CCA (same case as Fig. 1A). **B** Detection of E18-truncated *FGFR2* by measuring the ratio of the 5′ portion (exon 5; E5) versus the 3′ portion (E18) of the *FGFR2* gene. The ratio between the expressions of the E5 versus E18 of the FGFR2 gene ranged 0.42 to 32.00 (mean, 8.10) in FGFR2—immunohistochemically (IHC)—positive cHCC-CCAs and small duct iCCAs, whereas it ranged 0.06 to 8.94 (mean, 2.63) in FGFR2-IHC negative cases There was significant difference between FGFR2-positive and FGFR2-negative cases (*p* < 0.05)

*FGFR2::BICC1* fusion was detected in a case of cHCC-CCA (a 44-year-old female, glycogen storage disease type I, a tumor size of 7 cm, F4; same case as shown in Fig. 1A) (Fig. 2A). *FGFR2* fusions with other partners (*AHCYL1*, *PPHLN1*, *TACC2*, *CCDC6*, *MGEA5*, *G3BP2*, *OPTN*, *AFF3*, *CASP7*, *OFD1*, *KIAA1598*) were not detected in cHCC-CCAs and small duct-type iCCAs.

### Detection of E18-truncated FGFR2 in cHCC-CCAs and CCAs

Twenty-four cHCC-CCAs (17 FGFR-immunohistochemically (IHC)-positive and 7 FGFR-IHC negative cases) and 9 small duct-type iCCAs (8 FGFR-IHC-positive and one FGFR-IHC negative cases) were examined for the E18-truncated FGFR2 by measuring the ratio of the 5′ portion (E5) versus the 3′ portion (E18) of the FGFR2 gene expression. The ratio between the expressions of the E5 versus E18 of the FGFR2 gene ranged 0.42 to 32.00 (mean, 8.10) in FGFR2-IHC positive cHCC-CCAs and small duct iCCAs, whereas it ranged 0.06 to 8.94 (mean, 2.63) in FGFR2-IHC negative cases (Fig. 2B). The ratio between the expressions of the E5 versus E18 of the FGFR2 gene was more than 2 in 19 of 25 FGFR2-positive cHCC-CCAs and small duct iCCAs (76%) and 2 of 8 FGFR2-negative cases (25%). The 5′/3′ (E5/E18) imbalance in FGFR2 genes (E5/E18 ratio > 2) indicating E18-truncated FGFR2 was significantly more frequently detected in FGFR2-positive cHCC-CCAs and small duct iCCAs, compared to FGFR2-negative cases (*p* < 0.05) (Fig. 2B). The E5/E18 ratios in the FGFR-IHC-positive cases shown in Figs. 1A–C were 7.48, 22.7, and 20.6, respectively.

## Discussion

The data obtained in this study are summarized as follows: (1) FGFR2 expression was detected in significantly more patients with cHCC-CCA (21.3%) and small duct-type iCCA (25.7%), compared to those with large duct-type iCCA (3.3%) and HCC (0%) (*p* < 0.05); (2) FGFR2-positive cHCC-CCAs were significantly smaller size (*p* < 0.05), with more predominant cholangiolocarcinoma component (*p* < 0.01) and less nestin expression (*p* < 0.05). (3) Genetic alterations of ARID1A and BAP1 and multiple genes were significantly more frequent in FGFR2-positive cHCC-CCAs (*p* < 0.05). (4) *FGFR2::BICC1* fusion was detected in a case of cHCC-CCA with FGFR2 expression. (5) E18-truncated FGFR2 was significantly more frequently detected in FGFR2-positive cHCC-CCAs and small duct-type iCCAs, compared to FGFR2-negative ones (*p* < 0.05).

In the present study, we examined the immunohistochemical expression of FGFR2 as a surrogate marker for FGFR2 genetic alterations in cHCC-CCAs and other types of primary liver carcinomas. FGFR2-immunohistochemistry reportedly correlates with the FGFR2 genetic alterations, and it can be a surrogated marker with high specificity [18]. FGFR2 genetic alterations, especially FGFR2 fusions, were detected in 10–20% of iCCA, mainly in small duct-type iCCAs in previous studies [10–12, 15]. In the present study, FGFR2 expression was detected in 25.7% of small duct-type iCCAs, whereas FGFR2 expression was rarely detected in large duct-type iCCAs (3.3%). The prevalence rate and the selective detection of FGFR2 expression in small duct-type iCCAs are consistent with previous studies [10–12, 15]. These findings also support that the immunohistochemical FGFR2 expression is a good surrogate marker corresponding to FGFR2 genetic alterations.

In the present study, the FGFR2 expression was detected in 21.3% of cHCC-CCAs, similarly to small duct-type iCCAs. This finding clearly suggests that cHCC-CCAs with FGFR2 genetic alterations may be targets of the therapy with FGFR2 inhibitors, as well as small duct-type iCCAs. FGFR2 genetic alterations, especially FGFR2 fusions, were detected in 0–6.5% of cHCC-CCAs in previously [4–7]. Therefore, the frequency may be higher, compared to previous studies. The FGFR2 genetic alterations were more frequently detected in CCA-like cHCC-CCA than HCC-like cHCC-CCA [5]. In the present study, the FGFR2-positive cHCC-CCAs were significantly more frequent in cHCC-CCAs with predominant CLC component. Taken together, a higher proportion of CLC-component/CCA-like cHCC-CCA may be related to the higher frequency of FGFR2 expression in the present study. cHCC-CCA and small duct-type iCCA share various features, and a possible cell origin and a carcinogenesis pathway have been discussed [1, 2, 22]. FGFR2 genetic alterations may suggest one of such common features in cHCC-CCA and small duct-type iCCA.

There are several issues in terms of the sensitivity of the assays for FGFR2 genetic alterations using next-generation sequencing (NGS) or FISH [14], since over 150 genes have been identified as fusion partner with FGFR2 [12, 16]. We tried to detect several common FGFR2 fusions by using the FGFR2-fusion-specific primers. As results, *FGFR2::BICC1* fusion was detected in only one case of cHCC-CCA with FGFR2 expression in the present study. It is known that there are discrepancies between the FGFR2 fusions and effect of FGFR2 inhibitors [13, 21], which may be due to difficulties in the detection of diverse FGFR2 genetic alterations. More reliable assays may be mandatory for the detection of FGFR2 genetic alterations. Zingg et al. reported recently that E18-truncated variant of FGFR2 is a potent driver mutation, and any FGFR2 variant with a truncated E18 should be considered for FGFR-targeted therapies [17]. In the present study, we applied 5′/3′ imbalance RT-PCR for the detection of E18-truncated FGFR2 including FGFR2 fusion genes. E18-truncated FGFR2 was significantly more frequently detected in FGFR2-positive cHCC-CCAs and small duct-type iCCAs, compared to FGFR2-negative ones. These findings suggest that there may be other types of FGFR2 fusions which were not examined in this study in cHCC-CCAs and small duct-type iCCAs with FGFR2 expression. Taken together, the immunostaining and the PCR-based detection of FGFR2 genetic alterations may be useful surrogate markers for screening the application of FGFR2 inhibitors.

Interestingly, nestin expression was significantly lower in FGFR2-positive cHCC-iCCAs, compared to FGFR2-negative cHCC-iCCAs. Nestin, an embryonic type VI intermediate filament (IF) protein, was originally identified as a marker for neural stem cells in early development [6]. Recent studies revealed that cHCC-ICCs and small duct-type iCCAs showed the significantly higher expression of nestin, compared to HCCs [6, 22, 34, 35]. In our previous study [22], nestin-positive cHCC-CCA was characterized by a smaller tumor size, the more frequent presence of CLC components, a higher rate of p53 mutations, and a higher rate of multiple genetic alterations. In the present study, FGFR2-positive cHCC-CCAs were significantly smaller size, predominant CLC components and multiple genetic alterations, compared to FGFR2-negative cHCC-CCAs. Therefore, FGFR2-positive cHCC-CCAs and nestin-positive cHCC-CCAs share similar features such as smaller tumor size, the more frequent presence of CLC components, and multiple genetic alterations. There may be, however, some distinct difference between nestin-positive cHCC-CCAs and FGFR2-positive cHCC-CCAs.

The primary limitations of this study are the small cohort size and limited information on the association of the immunohistochemical FGFR2 expression with genetic alterations of FGFR2 and clinical outcomes. Analysis using NGS, especially RNA-based NGS, such as hybrid capture RNA NGS, is mandatory to further validate whether the immunohistochemical detection of FGFR2 expression is an effective surrogate marker for the detection of E18-truncated FGFR2 including FGFR2 fusion genes. If the immunohistochemical detection of FGFR2 expression is validated, the immunohistochemical assays may be used for screening the application of FGFR2 inhibitors. When FGFR2-immunohistochemistry was negative, further analysis using NGS would be applied. This strategy will be effective for shortening the turn-around time of NGS analysis and prompt application of FGFR2 inhibitors.

In conclusion, FGFR2 expression was detected in cHCC-CCAs as frequently as small duct-type iCCAs. This finding suggests a possible therapeutic indication of FGFR2 inhibitors for the patients with cHCC-CCAs.

### Supplementary Information

Below is the link to the electronic supplementary material.

Supplementary file1 (DOCX 30 KB)
