# Supplementary material for: Expression of fibroblast growth factor receptor 2 (FGFR2) in combined hepatocellular-cholangiocarcinoma and intrahepatic cholangiocarcinoma: clinicopathological study
Source: Virchows Arch. 2024 Mar 27;484(6):915–23. doi: 10.1007/s00428-024-03792-x (PMC11186861; doi:10.1007/s00428-024-03792-x)
Supplement: Supplementary file 1 — Supplementary file1 (DOCX 30 KB) [file 428_2024_3792_MOESM1_ESM.docx]

**Expression of fibroblast growth factor receptor 2 (FGFR2) in combined hepatocellular-cholangiocarcinoma and intrahepatic cholangiocarcinoma: Clinicopathological study**

Motoko Sasaki, M.D., PhD.^1^, Yasunori Sato, M.D., PhD ^1^ and Yasuni Nakanuma, M.D., PhD ^2^ 1) Department of Human Pathology, Kanazawa University Graduate School of Medical Sciences, Kanazawa, Japan; 2) Division of Pathology, Fukui Saiseikai Hospital, Fukui, Japan.

E-mail: [m8sasaki@med.kanazawa-u.ac.jp](mailto:m8sasaki@med.kanazawa-u.ac.jp)

**Supplementary Table 1. Primary antibodies used**

| ***Primary antibody*** | ***Type (clone)*** | ***Pre-treatment*** | ***dilution*** | ***Source*** |
| --- | --- | --- | --- | --- |
| FGFR2 | Rabbit mono (D4L2V) | eARI-BA (121℃, 5min) | 1:300 | Cell Signaling, Danvers, MA |
| ARID1A | Rabbit polyclonal | eARI-BA (121℃, 5min) | 1:300 | Sigma, St. Lois, MO |
| p53 | Mouse mono (DO7) | MW-CB (95℃, 20min) | 1:100 | Dako, Carpinteria, CA |
| PBRM1 | Rabbit polyclonal | eARI-BA (121℃, 5min) | 1: 200 | Bethyl, Montgomery, TX |
| BAP1 | Mouse mono (C-4) | eARI-BA (121℃, 5min) | 1: 100 | Santa-Cruz, Santa-Cruz, CA |
| MTAP | Mouse mono (2G4) | eARI-BA (121℃, 5min) | 1:300 | Abnova, Taipei,Taiwan |
| Nestin | Mouse mono (10C2) | MW-CB (95℃, 20min) | 1:200 | Santa-Cruz, Santa-Cruz, CA |

FGFR2, fibroblast growth factor receptor 2; ARID1A, AT-rich interactive domain-containing protein 1A; PBRM1, protein polybromo-1; BAP1, BRCA-associated protein 1; MTAP, methylthioadenosine phosphorylase; eARI, electronic antigen retrieval instrument; BA, 0.05M boric acid buffer (pH 8); MW, microwave; CB, 0.05M citric acid buffer (pH 6).

**Supplementary Table 2. PCR primers used**

| ***Gene*** | ***Region*** | ***Forward*** | ***Reverse*** |
| --- | --- | --- | --- |
| ***FGFR2-fusion*** |  |  |  |
| ***FGFR2***::***AHCYL1*** |  | 5’-CTCCCAGAGACCAACGTTCA | 5’-CTGTGAGATCGAGCGAGACA |
| ***FGFR2***::***BICC1*** |  | 5’-TGATGAGGGACTGTTGGCAT | 5’-TGGCCAAGCAATCTGCGTAT |
| ***FGFR2***::***BICC1*** |  | 5’-CTCCCAGAGACCAACGTTCA | 5’-TGGCCAAGCAATCTGCGTAT |
| ***FGFR2***::***PPHLN1*** |  | 5’-TGATGATGAGGGACTGTTGG | 5’-GGTGGTTTCTTTGGCACAAT |
| ***FGFR2***::***PPHLN1*** |  | 5’-CAGTTGGTAGAAGACTTGGATCG | 5’-GGTGGTTTCTTTGGCACAAT |
| ***FGFR2***::***TACC2*** |  | 5’-CTCCCAGAGACCAACGTTCA | 5’-TGGCCAAGCAATCTGCGTAT |
| ***FGFR2***::***CCDC6*** |  | 5’-CCAACGTTCAAGCAGTTGGT | 5’-TCTTCTTCCTGCTCAGCCCT |
| ***FGFR2::MGEA5*** |  | 5’-TGATGATGAGGGACTGTTG | 5’-GAGTTCCTTGTCACCATTTG |
| ***FGFR2***::***G3BP2*** |  | 5’-CTCCCAGAGACCAACGTTCA | 5’-ACAAACTCCCGCCCTACAAG |
| ***FGFR2***::***OPTN*** |  | 5’-TGATGATGAGGGACTGTTGG | 5’-GCCCAGGACTATGCTTGATT |
| ***FGFR2***::***AFF3*** |  | 5’-CCAACTGCACCAACGAACTG | 5’-GTGGAAGCCAGGTCATCTCC |
| ***FGFR2***::***CASP7*** |  | 5’-CCAGAGACCAACGTTCAAGC | 5’-GAATCCTCAACCCCCTGCTC |
| ***FGFR2***::***OFD1*** |  | 5’-CCAGAGACCAACGTTCAAGC | 5’-GAGGCTGCAGTTCTCCACTC |
| ***FGFR2***::***KIAA1598*** |  | 5’-CCAACGTTCAAGCAGTTGGT | 5’-CAGCTAACATGGACACTCTATTGC |
| ***3’/5’-imbalance*** |  |  |  |
| ***FGFR2*** | Exon 5 | 5’-TGGCTGAAAAACGGGAAGGA | 5’-AGTGCTGGTTTCGTACCTTGT |
|  | Exon 18 | 5’-TTCTCCAGACCCCATGCCTTA | 5’-AGGCAGACACAGTCATTCATGT |
| ***IDH1*** | Exon 4 | 5′-TCAGAGAAGCCATTATCTGCAAAAATAT | 5′-GGCCATGAAAAAAAAAACATGC |
| ***IDH2*** | Exon 4 | 5′- GGGTTCAAATTCTGGTTGAAAG | 5′-GGCGAGGAGCTCCAGTCG |
| ***KRAS*** | Exon1 | 5′-AGGCCTGCTGAAAATGACTG | 5′-ATCAAAGAATGGTCCTGCAC |
| ***BRAF*** | Exon15 | 5′-CATAATGCTTGCTCTGATAGGA | 5′-GGCCAAAAATTTAATCAGTGGA |
| ***GNAS*** | Exon 8 | 5′-ACTGTTTCGGTTGGCTTTGG | 5′-GGTAACAGTTGGCTTACTGG |
| ***TERT promoter*** | Chr 5 | 5′- CACCCGTCCTGCCCCTTCACCTT | 5′-GGCTTCCCACGTGCGCAGCAGGA |

FGFR2, fibroblast growth factor receptor 2; AHCYL1, adenosylhomocysteinase like 1; BICC1, BicC family RNA binding protein 1;

PPHLN1, periphilin 1; TACC2, transforming acidic coiled-coil containing protein 2; CCDC6, coiled-coil domain containing 6; MGEA5, meningioma expressed antigen 5; G3BP2, G3BP stress granule assembly factor 2; OPTN, optineurin; AFE3, ALF transcription elongation factor 3; CASP7, caspase 7; OFD1, OFD1 centriole and centriolar satellite protein; KIAA1598, KIAA1598/shootin-1; Chr, chromosome
